# Metastasis patterns and prognosis of octogenarians with metastatic breast cancer: A large-cohort retrospective study

**DOI:** 10.1371/journal.pone.0263104

**Published:** 2022-02-17

**Authors:** Zhenye Lv, Wendan Zhang, Yingjiao Zhang, Guansheng Zhong, Xiaofei Zhang, Qiong Yang, Ying Li

**Affiliations:** 1 Division of Breast Surgery, Department of General Surgery, Cancer Center, Zhejiang Provincial People’s Hospital, Affiliated People’s Hospital, Hangzhou Medical College, Hangzhou, Zhejiang, China; 2 Department of Gynaecology and Obstetrics, The 903 Hospital of the Joint Logistics Support Force of the Chinese People’s Liberation Army, Hangzhou, Zhejiang, China; 3 Department of Gastroenterology, The 903 Hospital of the Joint Logistics Support Force of the Chinese People’s Liberation Army, Hangzhou, Zhejiang, China; 4 Department of Breast Surgery, College of Medicine, The First Affiliated Hospital, Zhejiang University, Hangzhou, Zhejiang, China; Texas Tech University Health Science, Lubbock, UNITED STATES

## Abstract

**Background:**

Breast cancer may differ biologically in patients aged over 80 years. The objective of the current study was to analyze the metastasis patterns and prognosis of elderly patients with metastatic breast cancer (MBC) and compare it to patients of other ages.

**Methods:**

The Surveillance, Epidemiology, and End Results (SEER) database was utilized to select MBC patients from 2010 to 2015. Chi-squared test was used to compare clinicopathological characteristics among different aged groups. The Kaplan-Meier method and multivariate Cox model were utilized for survival analysis.

**Results:**

A total of 10479 MBC patients were included, among which 1036 (9.9%) patients were aged over 80 years. Compared with other aged group, the elderly patients tended to have a higher proportion of HR+/Her2- subtype, white race, lower tumor differentiation, and receive less treatment, including surgery, chemotherapy and radiotherapy (P< 0.001). MBC patients with different age presented with distinctive metastatic patterns. The older patients were more likely to have lung metastasis, but less likely to have bone, brain, liver and multiple sites metastasis than the younger group (P <0.001). The proportion of TNBC subtype increased substantially in the older patients with brain metastasis, compared to the younger and middle-aged group. The old age was demonstrated to significantly associate with worse prognosis of MBC patients. Additionally, our findings also showed that older MBC patients could achieve dramatical overall survival benefit from surgery (HR = 0.58; P <0.001) and chemotherapy (HR = 0.59; P <0.001), but not the radiotherapy (HR = 0.96; P = 0.097).

**Conclusion:**

The elderly MBC patients presented with distinctive metastatic patterns, clinical characteristics, and prognostic outcomes compared with younger patients. Our findings could assist clinicians in making appropriate therapeutic decision.

## Introduction

Breast cancer (BC) represents the most common malignancy in females, accounting for estimated 266120 new cases in the United States, in 2018 [1]. According to the latest statistical report, breast cancer still accounts for the second most common cause of death in females (14% of all cancer deaths) after lung cancer. It was reported that approximately 6% of breast cancer patients have been diagnosed with synchronous distant metastasis, such as bone, liver, lung and brain [2]. Although great advance has been made in the past several years, huge challenge still exists in patients with metastatic breast cancer (MBC), which is still associated with substantial morbidity and mortality. The median survival time of MBC patients is about 18–24 months, and the 5- and 10-year survival rates is as low as 27% and 13%, respectively [3].

It was reported that about 30–50% of breast cancer patients are aged over 65 years, among which patients over the age of 80 constitute a large proportion [4]. Approximately 25% of breast cancer patients over age of 65 or 10.6% of all BC patients is reported to be 80 years-old or older [5]. Moreover, study also showed that the incidence of breast cancer among the elderly group with 80–84 years-old (400 per 100,000 people) are much higher than that with 50–54 years-old (200 per 100,000 people) [6]. These older patients (≥80 years-old) often receive inferior screening for breast cancer, and present as an challenge in their treatment [7]. Due to comorbidities and frailty, the elderly patients are inevitably underrepresented in clinical trials. A previous key study revealed that only 9% of participants in clinical trial were aged over 75 years in comparison with 31% in the overall patients population [8]. Although oncologic indication exists, the decline of physiologic function and poor survival expectation make both elderly patients and doctors reluctant to pursue standard treatment recommended by clinical guideline, especially for those with distant metastasis [9]. Therefore, compared to the younger group, the disease may have distinctive biological characteristics in patients aged over 80 years old. So far, however, few previous reports have concentrated on analyzing the heterogeneity of metastatic pattern and prognosis among MBC patients older than 80 years.

In current study, the distant metastasis pattern and prognosis of elderly patients (≥80 years-old) were analyzed and compared to other age groups in a large cohort of metastatic breast cancer patients by using the SEER database.

## Methods

### Database and case selection

**Fig 1:**
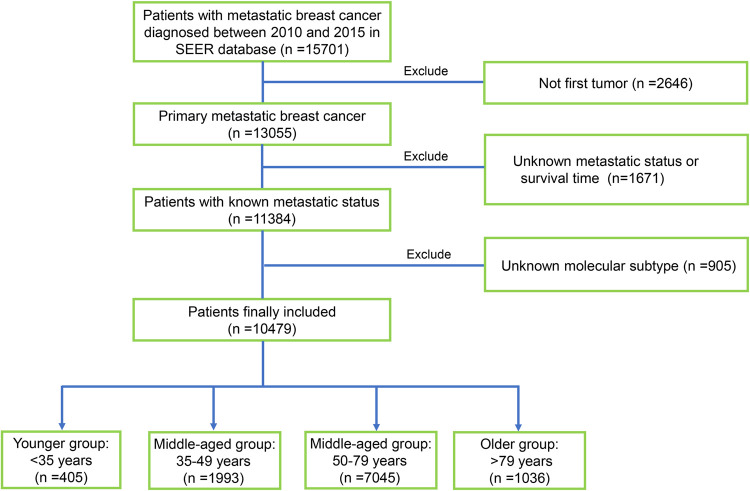
The flowchart of patients’ selection.

We performed a retrospective cohort research by utilizing the custom Surveillance, Epidemiology and End Results (SEER) database [Incidence- SEER 18 Regs Custom Data (with additional treatment fields), Nov 2018 Sub (1975–2016 varying)]. The SEER program, a database established by the National Cancer Institute of the U.S., collected data of cancer patients that accounts for about approximately 28% of the U.S. population [10]. The SEER*Stat software (version 8.3.8, National Cancer Institute, Washington, USA) was utilized to access the data from SEER database. Patients with de novo metastatic (M1) breast cancer (Site recode International Classification of Diseases for Oncology-3 (ICD-O-3)/WHO 2008:Breast) diagnosed in 2010 through 2015 were identified from this database. Only patients diagnosed with invasive ductal carcinoma (IDC) or invasive lobular carcinoma (ICC) as their first malignancy were eligible. In addition, patients with unknown follow-up, unknown molecular subtype, or unknown metastatic involvement, including bone, liver, lung, brain, and distant lymph node (DL) were excluded. Finally, a total of 10479 eligible patients were included in this study. It is well known that the cancer biology may quite different between young breast cancer patients and older patients. Recently, there is no international agreement on the definition of young breast cancer. Most of the literatures defined breast cancer patients younger than 35 or 40 years old as young breast cancer patients [11, 12]. Hence, based on the diagnosed age, patients in our study were subsequently divided into four groups: < 35 years old, 35–49 years old, 50–79 years old, and > 79 years old. The flowchart for the patient’s selection was shown in **Fig 1**.

### Covariates

Multiple variables were included in this study, including demographic characteristics (race and marital status), disease characteristics (AJCC T and N stage, histologic grade and molecular subtype), and treatment modalities (surgery, chemotherapy, and radiotherapy). Specially, races, including American Indians, AK Natives, Asians and Pacific Islanders, were classified into other races. Marital status, including divorced, separated, widowed, and domestic partner were categorized into other status. The main outcome were overall survival (OS) and cancer specific survival (CSS).

### Statistical analysis

Demographic and clinical characteristics between different cohorts were summarized by descriptive statistics and compared by using the Pearson’s Chi-square test or Fisher’s exact test. The Kaplan-Meier curves were plotted for the OS and CSS between different cohorts, and the Log Rank test for the comparison of difference among the curves. The multivariate logistic regression models were constructed to explore the association between the age and the sites of distant metastases. Subsequently, the univariate and multivariate Cox regression analyses were also conducted to identify the independent prognostic factors for the elder patients (> 79 years) with metastatic breast cancer.

Descriptive statistic, logistic regression analysis, and Cox proportional hazards analysis were performed by using the SPSS 24.0 (IBM Corp). The Kaplan-Meier curves and the forest plots were created by using the R software version 3.6.0. We defined a 2-sided P value of <0.05 as statistically significant unless otherwise stated.

### Ethics statement

The SEER database is an open database. Data released from the SEER database do not require informed patient consent, because cancer is a reportable disease in every state of the United States. The present study complied with the 1964 Helsinki Declaration and its later amendments or comparable ethical standards.

## Results

### Population characteristics

A total of 10479 metastatic breast cancer (MBC) patients diagnosed from 2010 to 2015 were finally extracted from the SEER database, among which 1036 (9.9%) patients were at the age of ≥ 80 years old, 7045 (67.2%) patients within 50–79 years old, 1993 (19.0%) patients within 35–49 years old, and 405 (3.9%) patients under 35 years old. In comparison with younger (< 35 yrs) and middle-aged (35–49 or 50–79 yrs) patients, the older patients (≥ 80 yrs) had a higher rate of white race (84.6% vs. 62.5% vs. 68.8% vs. 74.2%, respectively, P <0.001), and higher rate of histologic grade 1–2 (48.4% vs. 33.1% vs. 39.0% vs. 44.1%, respectively, P <0.001). On the contrary, compared to younger and middle-aged patients, the older patients exhibited a lower rate of lymph node metastasis (28.1% vs. 14.6% vs. 16.8% vs. 20.8% for N0, respectively, P <0.001), and received less treatment, such as surgery (77.5% vs. 55.1% vs. 58.8% vs. 66.8%, respectively, P <0.001), radiotherapy (77.6% vs. 56.8% vs. 57.3% vs. 64.6%, respectively, P <0.001), and chemotherapy (81.7% vs. 13.6% vs. 23.8% vs. 42.4%, respectively, P <0.001). Regarding to the molecular subtype, specifically, the older MBC patients had a higher rate of type of HR+/Her2- (67.7% vs. 40.7% vs. 53.6% vs. 61.3%, respectively, P <0.001), but lower rate of HR+/Her2+ (13.0% vs. 27.9% vs. 20.7% vs. 16.5%, respectively, P <0.001) and HR-/Her2+ (5.9% vs. 17.8% vs. 10.7% vs. 9.0%, respectively, P <0.001), when compared to other aged groups. The detailed information for clinicopathological features among different aged groups were showed in **Table 1**.

**Table 1:** Characteristics of patients with metastatic breast cancer by age groups.

Characteristic	Age<35 years	35–49 years	50–79 years	Age>79 years	Total	P value
	No. (%)	No. (%)	No. (%)	No. (%)	No. (%)	
Race						< .001
Black	104 (25.7)	416 (20.9)	1207 (17.1)	111 (10.7)	1838 (17.5)	
White	253 (62.5)	1372 (68.8)	5226 (74.2)	876 (84.6)	7727 (73.7)	
Others^a^	48 (11.9)	195 (9.8)	590 (8.4)	46 (4.4)	879 (8.4)	
Unknown	0 (0)	10 (0.5)	22 (0.3)	3 (0.3)	35 (0.3)	
Marital status						< .001
Single	171 (42.2)	577 (29.0)	1469 (20.9)	94 (9.1)	2311 (22.1)	
Married	190 (46.9)	1068 (53.6)	3183 (45.2)	227 (21.9)	4668 (44.5)	
Others^b^	23 (5.7)	269 (13.5)	2010 (28.5)	648 (62.5)	2950 (28.2)	
Unknown	21 (5.2)	79 (4.0)	383 (5.4)	67 (6.5)	550 (5.2)	
Histologic type						< .001
Invasive ductal carcinoma	393 (97.0)	1830 (91.8)	6042 (85.8)	868 (83.8)	9133 (87.2)	
Invasive lobular carcinoma	12 (3.0)	163 (8.2)	1003 (14.2)	168 (16.2)	1346 (12.8)	
Histologic grade						< .001
1–2	134 (33.1)	777 (39.0)	3106 (44.1)	501 (48.4)	4518 (43.1)	
3–4	243 (60.0)	1001 (50.2)	3038 (43.1)	394 (38.0)	4676 (44.6)	
Unknown	28 (6.9)	215 (10.8)	901 (12.8)	141 (13.6)	1285 (12.3)	
T stage						< .001
T1	40 (9.9)	204 (10.2)	763 (10.8)	108 (10.4)	1115 (10.6)	
T2	133 (32.8)	691 (34.7)	2112 (30.0)	343 (33.1)	3279 (31.3)	
T3	97 (24.0)	377 (18.9)	1111 (15.8)	145 (14.0)	1730 (16.5)	
T4	109 (26.9)	558 (28.0)	2342 (33.2)	314 (30.3)	3323 (31.7)	
Tx	26 (6.4)	163 (8.2)	717 (10.2)	126 (12.2)	1031 (9.8)	
N stage						< .001
N0	59 (14.6)	334 (16.8)	1465 (20.8)	291 (28.1)	2149 (20.5)	
N1	203 (50.1)	991 (49.7)	3176 (45.1)	431 (41.6)	4801 (45.8)	
N2	52 (12.8)	246 (12.3)	863 (12.2)	99 (9.6)	1260 (12.0)	
N3	78 (19.3)	329 (16.5)	1110 (15.8)	106 (10.2)	1623 (15.5)	
Nx	13 (3.2)	93 (4.7)	431 (6.1)	109 (10.5)	646 (6.2)	
Molecular subtype						< .001
HR+/Her2-	165 (40.7)	1068 (53.6)	4317 (61.3)	701 (67.7)	6251 (59.7)	
HR+/Her2+	113 (27.9)	412 (20.7)	1164 (16.5)	135 (13.0)	1824 (17.4)	
HR-/Her2+	72 (17.8)	214 (10.7)	631 (9.0)	61 (5.9)	978 (9.3)	
Triple Negative	55 (13.6)	299 (15.0)	933 (13.2)	139 (13.4)	1426 (13.6)	
Surgery						< .001
No	223 (55.1)	1171 (58.8)	4703 (66.8)	803 (77.5)	6900 (65.8)	
Yes	182 (44.9)	822 (41.2)	2342 (33.2)	233 (22.5)	3579 (34.2)	
Radiotherapy						< .001
No	230 (56.8)	1141 (57.3)	4548 (64.6)	804 (77.6)	6723 (64.2)	
Yes	175 (43.2)	852 (42.7)	2497 (35.4)	232 (22.4)	3756 (35.8)	
Chemotherapy						
No	55 (13.6)	474 (23.8)	2986 (42.4)	846 (81.7)	4361 (41.6)	
Yes	350 (86.4)	1519 (76.2)	4059 (57.6)	190 (18.3)	6118 (58.4)	

^a^ Other races included American Indians, AK Natives, Asians and Pacific Islanders.

^b^ Other status including divorced, widowed, separated or domestic partner.

### Metastasis pattern

**Fig 2:**
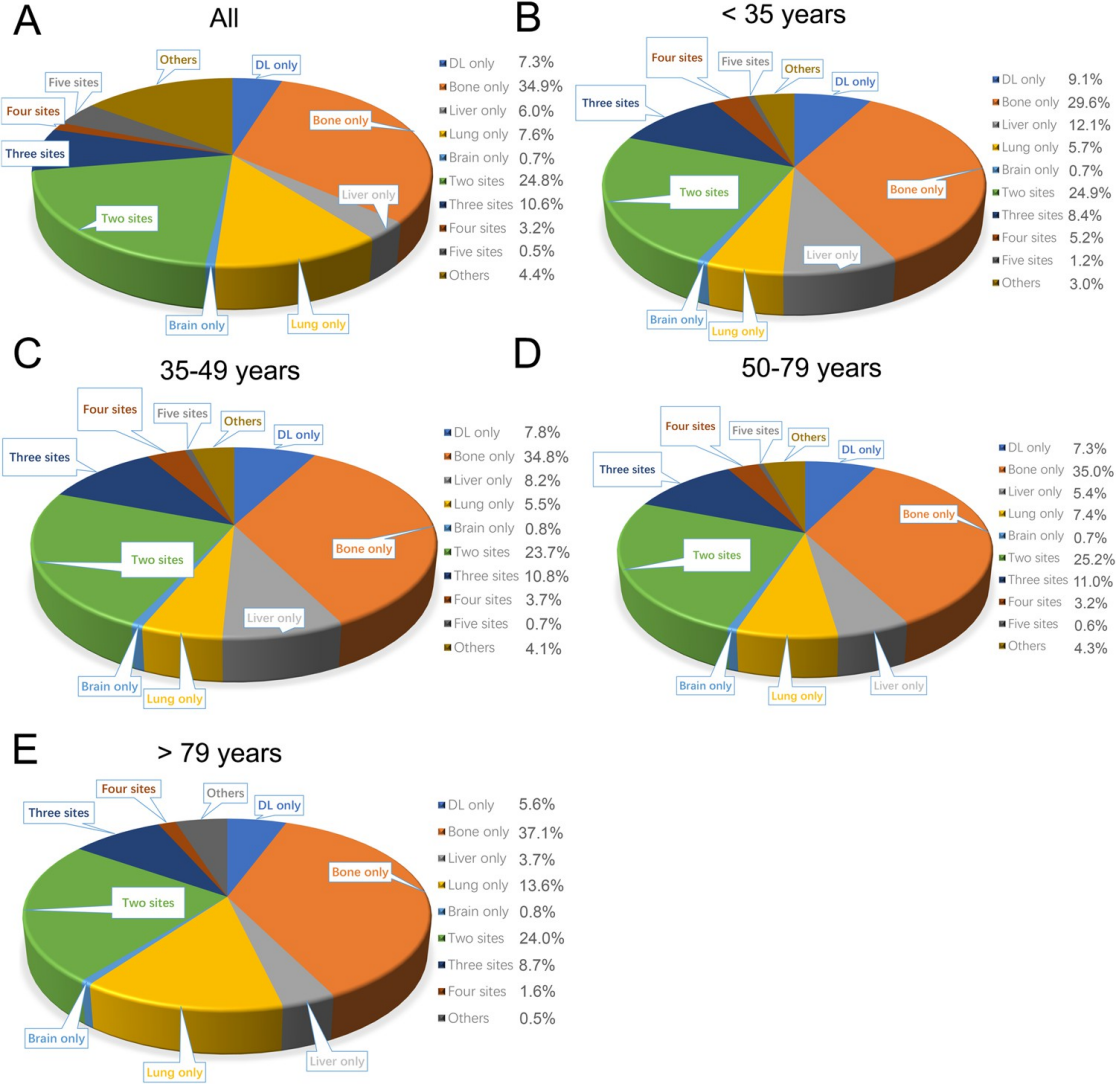
Distribution of single-organ and multi-organ metastatic sites in MBC patients with different age. (**A**) All patients; (**B**) Younger patients (<35 yrs); (**C**) Middle-aged patients (35–49 yrs); (**D**) Middle-aged patients (50–79 yrs); (**E**) Older patients (> 79 yrs).

**Fig 3:**
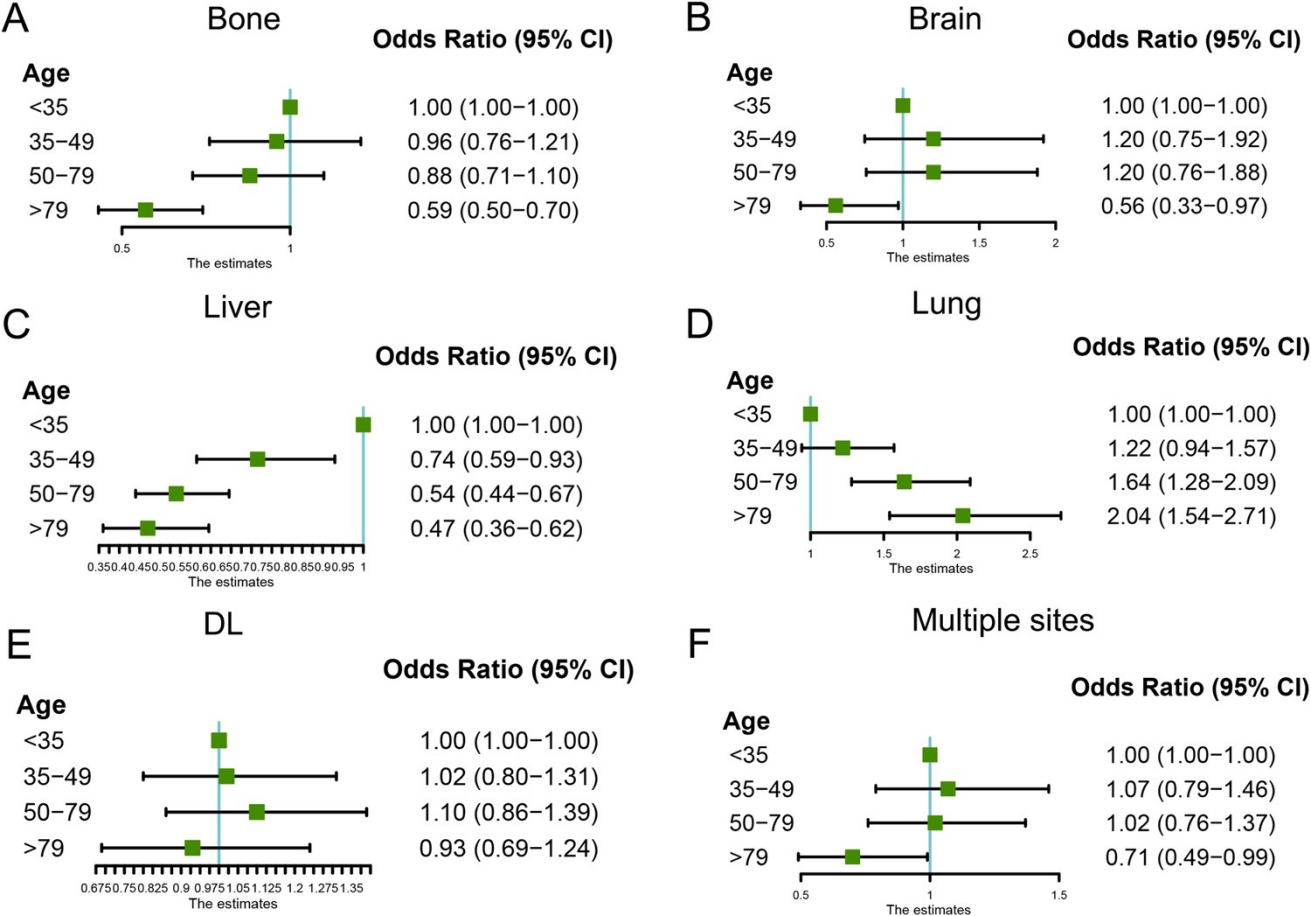
Multivariable logistic regression analyses predicting different metastasis sites in MBC patients. (**A**) Bone metastasis; (**B**) Brain metastasis; (**C**) Liver metastasis; (**D**) Lung metastasis; (**E**) DL metastasis; (**F**) Multiple sites metastasis. DL, Distant Lymph node.

In the whole cohort of MBC patients, the most common single metastases site was bone (34.9%), followed by lung only (7.6%), DL only (7.3%), liver only (6.0%), and brain only (0.7%), respectively (**Fig 2A**). 24.8% of patients were diagnosed with two distant metastasis sites, while 14.0% of patients had three or more metastases sites. In addition, the metastatic patterns between different aged groups were also compared. As shown in **Fig 2** and **Table 2**, the older group had the highest incidence of lung (13.5% vs. 0.2% vs. 10.5% vs. 4.9%, respectively, P <0.001) metastasis only and the lowest incidence of liver (0.4% vs. 0.5% vs. 1.6% vs. 3.6%, respectively, P <0.001) compared with the younger and middle-aged groups. Subsequently, we further performed a multivariate logistic analysis that adjusted for various confounding variables, including race, marital status, histologic type, histologic grade, AJCC T and N stage, molecular subtype, and therapies. The results indicated that the older group tended to have less bone metastasis (OR = 0.59; 95% CI [0.50–0.70]; P <0.001), brain metastasis (OR = 0.56; 95% CI [0.33–0.97]; P = 0.014), liver metastasis (OR = 0.47; 95% CI [0.36–0.62]; P <0.001), and multiple sites metastasis (OR = 0.71; 95% CI [0.49–0.99]; P = 0.041) than the younger group (**Fig 3**). On the contrary, relative to the younger group, the older group was more likely to occur lung metastasis (OR = 2.04; 95% CI [1.54–2.71]; P <0.001) than the younger group (**Fig 3D**).

**Table 2:** Comparison of organ metastasis patterns of patients with metastatic breast cancer by age groups.

Parameter	Age<35 years	35–49 years	50–79 years	Age >79 years	Total	P value
	No. (%)	No. (%)	No. (%)	No. (%)	No. (%)	
One site						
Bone only	120 (1.2)	693 (6.6)	2464 (23.5)	384 (3.7)	3662 (34.9)	0.125
Lung only	23 (0.2)	110 (10.5)	518 (4.9)	141 (13.5)	792 (7.6)	<0.001
Liver only	49 (0.5)	164 (1.6)	377 (3.6)	38 (0.4)	628 (6.0)	<0.001
Brain only	3 (0.03)	16 (0.2)	51 (0.5)	9 (0.08)	79 (0.7)	0.673
DL only	37 (0.4)	155 (1.5)	516 (4.9)	58 (0.5)	766 (7.3)	0.006
Multiple sites	161 (1.5)	774 (7.4)	2814 (26.9)	356 (3.4)	4105 (39.2)	0.002

DL, distant lymph node.

**Fig 4:**
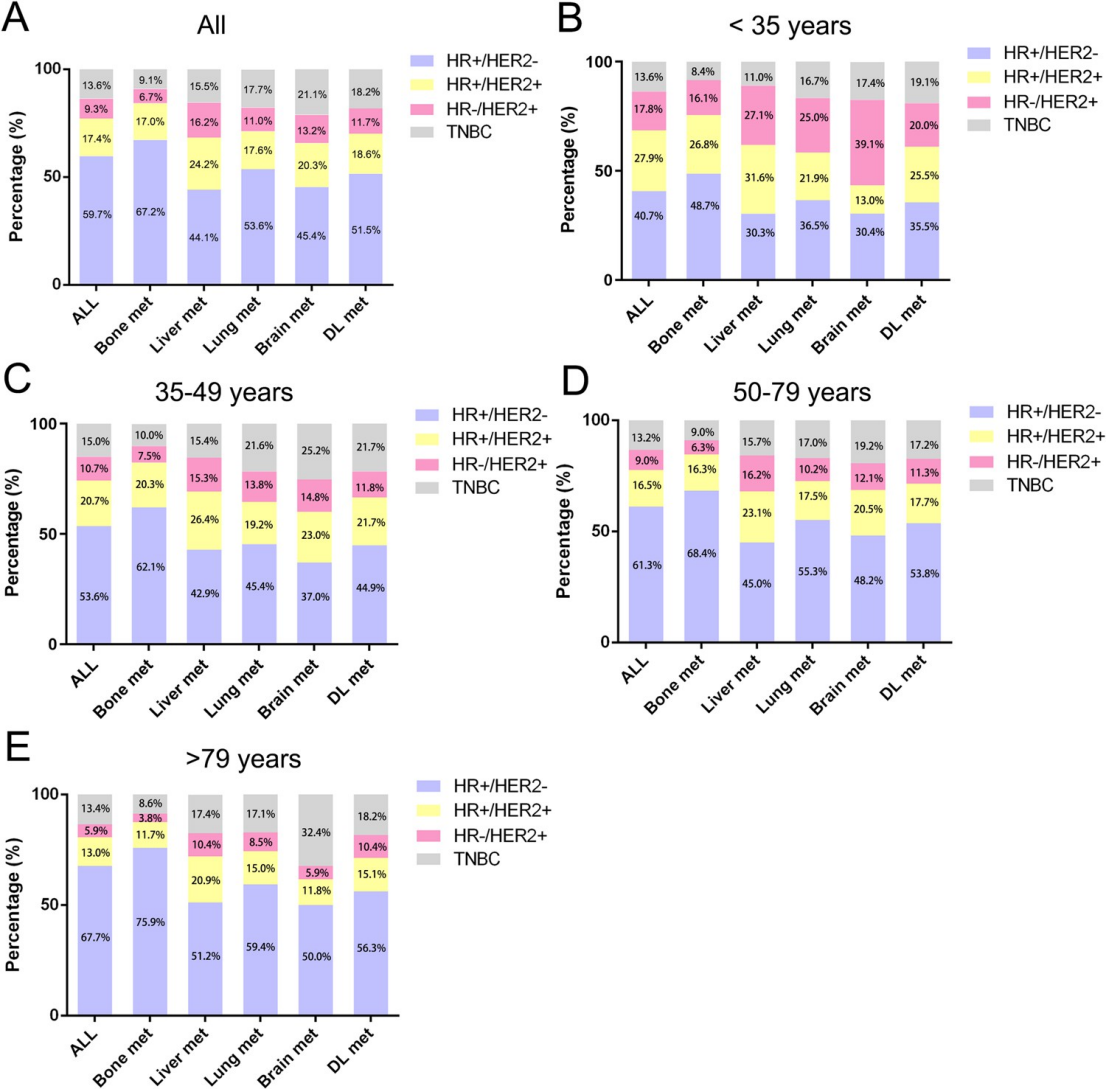
Distribution of molecular subtypes in MBC patients with different age. (**A**) All patients; (**B**) Younger patients (<35 yrs); (**C**) Middle-aged patients (35–49 yrs); (**D**) Middle-aged patients (50–79 yrs); (**E**) Older patients (> 79 yrs).

The distribution of molecular subtypes in patients with specific metastatic site was further investigated (**Fig 4**). The older patients with bone metastasis had the highest proportion (75.9%) of HR+/HER2- subtype compared with those who had other metastatic sites. The similar trends were also found both in the younger (48.7%) and the middle-aged (62.1% or 68.4%) group. Our result also indicated that the proportion of TNBC subtype increased in patients with visceral metastases, especially for brain metastasis. Additionally, it was noted that the proportion of TNBC subtype increased most in the older patients (32.4%) with brain metastasis, compared to the younger (17.4%) and the middle-aged (25.2% or 19.2%) patients.

### Survival

**Fig 5:**
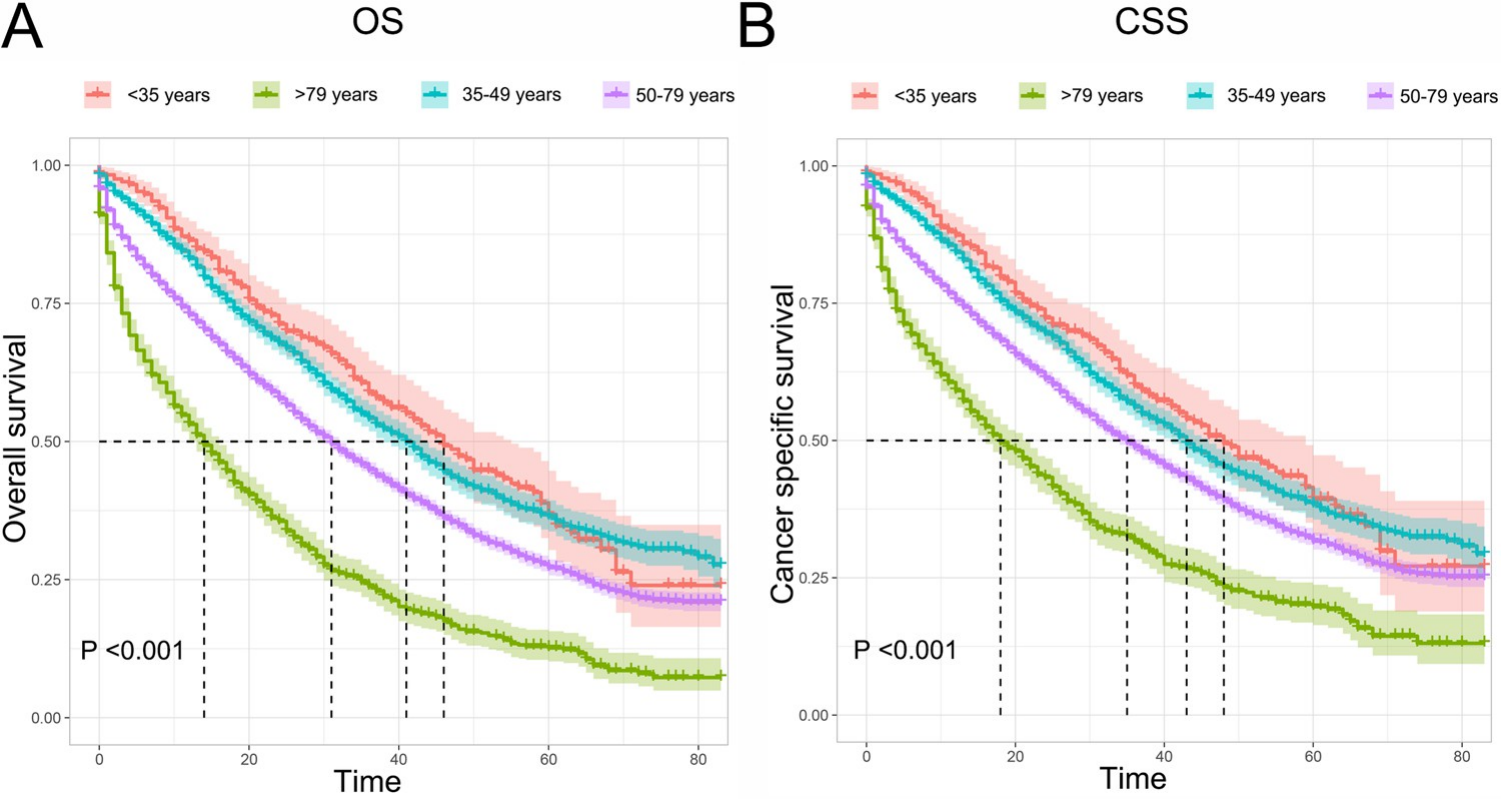
Kaplan-Meier curve of OS (**A**) and CSS (**B**) in MBC patients with different age. OS, overall survival; CSS, Cancer-specific survival.

**Fig 6:**
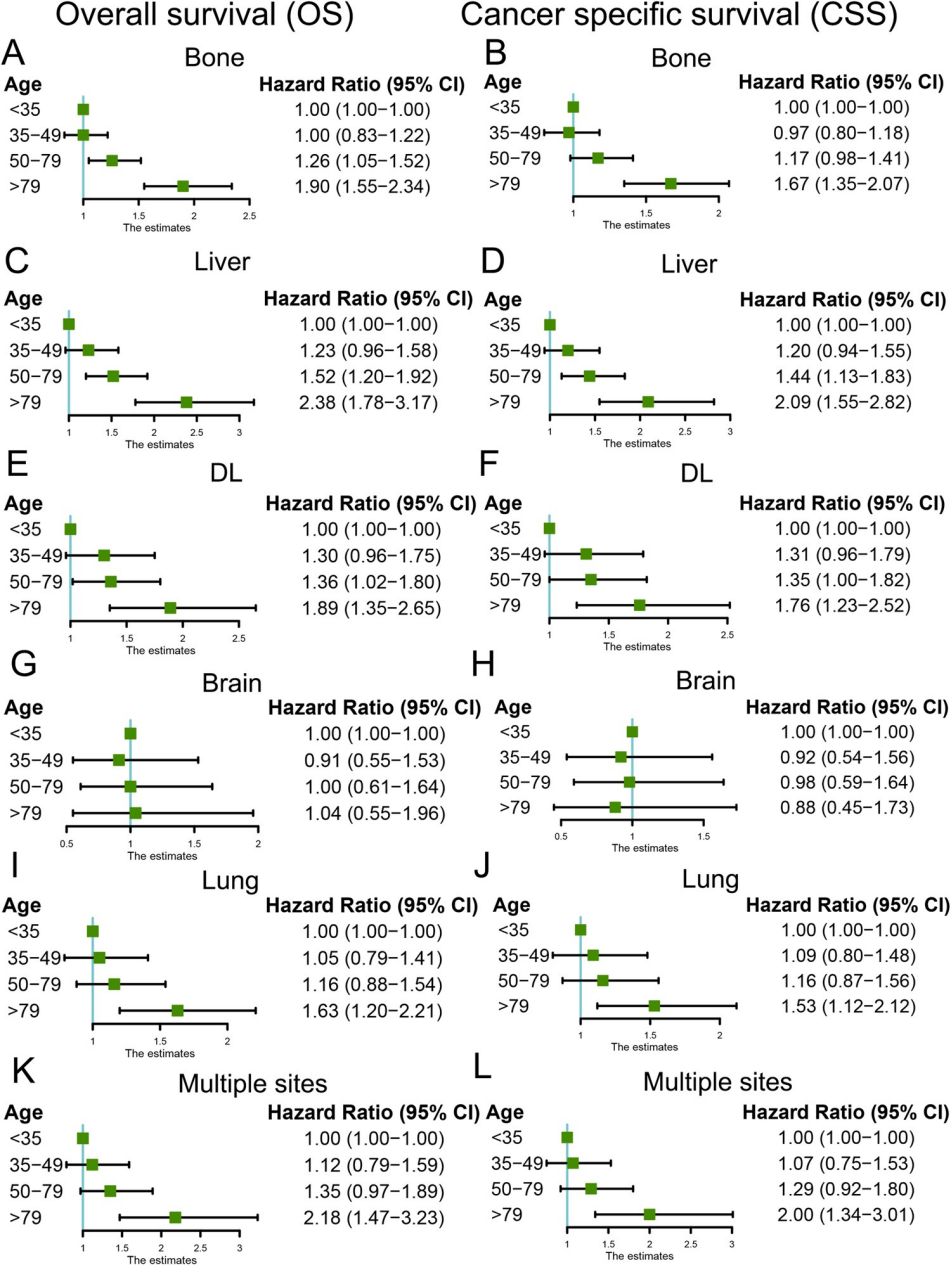
Multivariate Cox regression analyses for OS and CSS in MBC patients with different metastasis sites. (**A**) OS in patients with bone metastasis; (**B**) CSS in patients with bone metastasis; (**C**) OS in patients with liver metastasis; (**D**) CSS in patients with Liver metastasis; (**E**) OS in patients with DL metastasis; (**F**) CSS in patients with DL metastasis; (**G**) OS in patients with brain metastasis; (**H**) CSS in patients with brain metastasis; (**I**) OS in patients with lung metastasis; (**J**) CSS in patients with lung metastasis; (**K**) OS in patients with multiple sites metastasis; (**L**) CSS in patients with multiple sites metastasis. OS, overall survival; CSS, Cancer-specific survival; DL, Distant Lymph node.

Both of the OS and CSS between the four age groups were compared in this study. The Kaplan-Meier curves showed that the older group had the shortest OS and BCSS (P <0.001), with the median survival time being 13 (mean, 17.5 months), 26 (mean, 29.9 months), 27 (mean, 30.3 months) and 22 (mean, 25.8 months) months in the older, younger and middle-aged group, respectively (**Fig 5**). After adjusted for various confounding variables, the multivariate Cox analysis also confirmed that age was an independent prognostic factor. Compared to the younger group, the older age was significantly associated with worse OS (HR = 2.11; 95% CI [1.78–2.50]; P <0.001) and CSS (HR = 1.89; 95% CI [1.58–2.26]; P <0.001) (**S1 Table**). Moreover, expect for MBC patients with brain metastases, the older group showed poorer OS and CSS than the younger group in all subgroup stratified by different metastasis sites, including bone, lung, liver, DL, and multiple metastasis sites (**Fig 6**).

We then performed univariate and multivariate Cox analysis to explore prognostic factors that associated with OS (**Table 3**) and CSS (**Table 4**) for older patients with metastasis breast cancer. The multivariate analysis, as shown in **Table 4**, showed that patients with white race (vs black; HR = 0.87; 95% CI [0.82–0.94]; P <0.001), married (vs single; HR = 0.82; 95% CI [0.76–0.88]; P <0.001), N1 (vs N0; HR = 0.81; 95% CI [0.85–0.98]; P = 0.010), molecular subtype HR+/Her2+ (vs HR+/Her2-; HR = 0.81; 95% CI [0.75–0.88]; P <0.001) were significantly associated with increased CSS of older MBC patients. On the contrary, patients who had clinicopathological factors like histologic grade 3–4 (vs grade 1–2; HR = 1.39; 95% CI [1.30–1.48]; P <0.001), T2 (vs T1; HR = 1.12; 95% CI [1.01–1.24]; P = 0.030), T3 (vs T1; HR = 1.22; 95% CI [1.09–1.36]; P = 0.001), T4 (vs T1; HR = 1.40; 95% CI [1.27–1.56]; P <0.001), TNBC subtype (vs HR+/Her2-; HR = 2.91; 95% CI [2.68–3.16]; P <0.001), bone metastasis (vs DL metastasis; HR = 1.15; 95% CI [1.02–1.31], liver metastasis (vs DL metastasis; HR = 1.52; 95% CI [1.29–1.79]; P <0.001), lung metastasis (vs DL metastasis; HR = 1.27; 95% CI [1.09–1.48]; P = 0.002), brain metastasis (vs DL metastasis; HR = 2.46; 95% CI [1.83–3.30]; P <0.001), multiple sites metastasis (vs DL metastasis; HR = 1.98; 95% CI [1.76–2.24]; P <0.001) were dramatically associated with decreased CSS. Regarding to the treatment modalities, older MBC patients who received surgery (HR = 0.57; 95% CI [0.53–0.61]; P <0.001) or chemotherapy (HR = 0.62; 95% CI [0.58–0.66]; P <0.001) had dramatically favorable CSS in comparison with those received no treatment. A similar survival trend was also observed for OS (**Table 3**).

**Table 3:** Univariate analysis and multivariate analysis for overall survival (OS) among patients with metastatic breast cancer.

Variables	Univariate analysis		Multivariate analysis	
	HR (95% CI)	P value	HR (95% CI)	P value
Race		< .001		
Black	Reference		Reference	
White	0.76 (0.71–0.81)	< .001	0.86 (0.80–0.92)	< .001
Others^a^	0.70 (0.63–0.78)	< .001	0.78 (0.70–0.87)	< .001
Unknown	0.40 (0.21–0.77)	< .001	0.41 (0.22–0.80)	0.008
Marital status		< .001		
Single	Reference		Reference	
Married	0.76 (0.71–0.81)	< .001	0.82 (0.77–0.88)	< .001
Others^b^	1.17 (1.09–1.25)	< .001	1.13 (1.05–1.21)	0.001
Unknown	0.98 (0.87–1.10)	0.706	0.94 (0.84–1.06)	0.334
Histologic type				
Invasive ductal carcinoma	Reference			
Invasive ductal carcinoma	1.01 (0.94–1.09)	0.796		
Histologic grade		< .001		
1–2	Reference		Reference	
3–4	1.38 (1.30–1.45)	< .001	1.35 (1.27–1.44)	< .001
Unknown	1.35 (1.24–1.46)	< .001	1.14 (1.05–1.24)	0.002
T stage		< .001		
T1	Reference		Reference	
T2	1.08 (0.99–1.19)	0.099	1.12 (1.02–1.24)	0.020
T3	1.27 (1.14–1.41)	< .001	1.21 (1.09–1.35)	< .001
T4	1.60 (1.45–1.75)	< .001	1.40 (1.27–1.54)	< .001
Tx	1.62 (1.45–1.81)	< .001	1.31 (1.16–1.47)	< .001
N stage		< .001		
N0	Reference		Reference	
N1	0.93 (0.87–0.99)	0.022	0.89 (0.83–0.95)	0.001
N2	0.88 (0.80–0.97)	0.007	0.97 (0.89–1.07)	0.583
N3	0.93 (0.86–1.02)	0.109	0.95 (0.87–1.04)	0.268
Nx	1.46 (1.31–1.62)	< .001	1.19 (1.07–1.32)	0.002
Molecular subtype		< .001		
HR+/Her2-	Reference		Reference	
HR+/Her2+	0.75 (0.70–0.81)	< .001	0.81 (0.75–0.88)	< .001
HR-/Her2+	0.96 (0.87–1.05)	0.330	1.09 (0.98–1.20)	0.112
Triple Negative	2.37 (2.22–2.53)	< .001	2.84 (2.63–3.06)	< .001
Metastasis site		< .001		
DL	Reference		Reference	
Bone	1.09 (0.97–1.22)	0.152	1.10 (0.98–1.24)	0.111
Liver	1.32 (1.14–1.53)	< .001	1.47 (1.26–1.71)	< .001
Lung	1.47 (1.28–1.68)	< .001	1.21 (1.05–1.39)	0.009
Brain	2.98 (2.26–3.93)	< .001	2.32 (1.75–3.06)	< .001
Multiple sites	1.99 (1.78–2.22)	< .001	1.84 (1.64–2.06)	< .001
Surgery				
No	Reference		Reference	
Yes	0.54 (0.51–0.57)	< .001	0.58 (0.54–0.61)	< .001
Radiotherapy				
No	Reference		Reference	
Yes	0.81 (0.77–0.86)	< .001	0.96 (0.90–1.01)	0.097
Chemotherapy				
No	Reference		Reference	
Yes	0.67 (0.63–0.70)	< .001	0.59 (0.56–0.63)	< .001

Abbreviations: CI: confidence interval; HR: Hazard ratio.

^a^ including American Indians, AK Natives, Asians and Pacific Islanders.

^b^ including divorced, widowed, separated or domestic partner.

**Table 4:** Univariate analysis and multivariate analysis for cancer specific survival (CSS) among patients with metastatic breast cancer.

	Univariate analysis		Multivariate analysis	
Variables	HR (95% CI)	P value	HR (95% CI)	P value
Race		< .001		
Black	Reference		Reference	
White	0.77 (0.72–0.82)	< .001	0.87 (0.82–0.94)	< .001
Others^a^	0.71 (0.63–0.79)	< .001	0.80 (0.71–0.89)	< .001
Unknown	0.30 (0.14–0.67)	0.003	0.32 (0.14–0.72)	0.006
Marital status		< .001		
Single	Reference		Reference	
Married	0.76 (0.71–0.81)	< .001	0.82 (0.76–0.88)	< .001
Others^b^	1.12 (1.04–1.21)	0.002	1.09 (1.01–1.17)	0.028
Unknown	0.95 (0.84–1.08)	0.422	0.92 (0.81–1.05)	0.198
Histologic type				
Invasive ductal carcinoma	Reference			
Invasive ductal carcinoma	1.03 (0.95–1.11)	0.482		
Histologic grade		< .001		
1–2	Reference		Reference	
3–4	1.43 (1.35–1.52)	< .001	1.39 (1.30–1.48)	< .001
Unknown	1.39 (1.28–1.51)	< .001	1.18 (1.08–1.28)	< .001
T stage		< .001		
T1	Reference		Reference	
T2	1.09 (0.98–1.21)	0.099	1.12 (1.01–1.24)	0.030
T3	1.29 (1.15–1.44)	< .001	1.22 (1.09–1.36)	0.001
T4	1.62 (1.47–1.79)	< .001	1.40 (1.27–1.56)	< .001
Tx	1.60 (1.42–1.81)	< .001	1.30 (1.15–1.47)	< .001
N stage		< .001		
N0	Reference		Reference	
N1	0.96 (0.89–1.03)	0.253	0.91 (0.85–0.98)	0.010
N2	0.90 (0.82–0.99)	0.033	0.98 (0.89–1.09)	0.760
N3	0.96 (0.88–1.05)	0.405	0.97 (0.88–1.06)	0.471
Nx	1.42 (1.27–1.59)	< .001	1.16 (1.03–1.30)	0.015
Molecular subtype		< .001		
HR+/Her2-	Reference		Reference	
HR+/Her2+	0.77 (0.71–0.84)	< .001	0.81 (0.75–0.88)	< .001
HR-/Her2+	0.97 (0.88–1.07)	0.535	1.07 (0.96–1.19)	0.238
Triple Negative	2.49 (2.32–2.67)	< .001	2.91 (2.68–3.16)	< .001
Metastasis site		< .001		
DL	Reference		Reference	
Bone	1.11 (0.98–1.25)	0.103	1.15 (1.02–1.31)	0.029
Liver	1.36 (1.16–1.59)	< .001	1.52 (1.29–1.79)	< .001
Lung	1.52 (1.31–1.76)	< .001	1.27 (1.09–1.48)	0.002
Brain	3.13 (2.34–4.19)	< .001	2.46 (1.83–3.30)	< .001
Multiple sites	2.12 (1.88–2.39)	< .001	1.98 (1.76–2.24)	< .001
Surgery				
No	Reference		Reference	
Yes	0.54 (0.51–0.57)	< .001	0.57 (0.53–0.61)	< .001
Radiotherapy				
No	Reference		Reference	
Yes	0.83 (0.78–0.88)	< .001	0.98 (0.92–1.03)	0.390
Chemotherapy				
No	Reference		Reference	
Yes	0.71 (0.67–0.75)	< .001	0.62 (0.58–0.66)	< .001

Abbreviations: CI: confidence interval; HR: Hazard ratio.

^a^ including American Indians, AK Natives, Asians and Pacific Islanders.

^b^ including divorced, widowed, separated or domestic partner.

## Discussion

In current study, we systematically analyzed the distant metastasis patterns of MBC patients aged over 80 years and compared it to younger or middle-aged counterparts. Our results indicated that the elderly patients had distinctive clinicopathological characteristics and metastasis patterns. Our data also demonstrated that age at diagnosis was an independent risk factor for distant organ metastasis. Additionally, various independent prognostic factors were also identified for MBC patients aged over 80 years.

The proportion of elderly MBC patients (≥ 80 yrs) was 9.9% in current study, which echoed previously published studies implying that octogenarians represent approximately one in ten BC patients [5]. Our data found that the elderly MBC patients had a higher proportion of white race than younger patients, which might be due to genetic heterogeneity or social and economic factors. We also found that the older patients had a higher proportion of HR+/Her2- subtype but a lower proportion of other molecular subtypes, including HR+/Her2+ and HR-/Her2+. These data were inconsistent with the results of previous study that showed a similar molecular subtype between octogenarian patients and patients with 60–70 years old, which partly due to its small number of cases [13]. Recently, accumulating evidence have demonstrated that the expression level of HER-2 is positively related to the lymph node involvement and malignant behavior of breast cancer [14–16]. These findings might partly explain the current data indicating that the older MBC patients had lower histological grade and less lymph node involvement than younger patients.

Regarding the metastasis pattern in this study, the most and least frequent metastasis lesion were bone and brain, respectively. Our result was consistent with previous studies [17, 18]. Purushotham, et al. suggested a striking relationship between increasing age at diagnosis and decreased risk of distant metastasis to both bone and viscera in breast cancer patients [19]. Consistent to this study, our data further demonstrated that the elderly patients were less likely to have distant bone, brain, liver, lymph node, and multiple sites metastasis than the younger counterparts. Aging-related deterioration of the immune system, also called ‘immuno-senescence’, might partially account for the phenomenon [20]. Recent researches have demonstrated that co-opting the immune system is an important part of the metastatic process [21]. Hence, we hypothesized that immuno-senescence accompanied by advancing age may act as protective factor for distant metastasis through depriving the key immune-cellular components associated with metastatic process. However, as an exception, we also found that the older group had a higher risk of lung metastasis than the younger group. One possible factor contributing to this phenomenon would be aging-related low-grade inflammation, which was reported to facilitate tumor progression [22, 23]. Moreover, Wculek, et al. also reported that an altered presence of neutrophils contribute to lung colonization of metastasis-initiating breast cancer cells [24]. Additionally, the relationship between molecular subtype and metastasis were further studied in our study. Our data indicated a distinctive proportion of molecular subtype in different aged patients with different metastasis lesion. The proportion of HER2+ and TNBC subtype increased in patients with visceral metastases, which echoes previous study suggesting that ER-/PR- and/or HER2+ were more likely to metastasize to viscera compared to the reference category [19]. Additionally, consistent to previous researches [25–27], our study also indicated that patients with brain metastases were more likely to be TNBC subtype, especially in the older group. This result implied that the genetic alteration in different aged group as well as different molecular subtype may associated with increased risk of metastasis to specific organs. Moreover, the ‘seed and soil’ hypothesis might also a possible contributor [28]. The underlying molecular mechanisms still need to be further investigated.

In our survival analysis, our Kaplan-Meier curve as well as the multivariable Cox model demonstrated that older age dramatically contributed to worse OS and CSS. Moreover, in almost all subgroup with different metastasis site, the old aged patients had the worst survival in comparison with the younger group. This phenomenon might be explained by multiple factors, such as decreased physiologic function, increased risk of non-cancer death, and reduced treatment willingness. Supporting this viewpoint are previous reports showing that elderly patients are often not only offered less surgical treatment but also received less postoperative adjuvant chemotherapy and radiotherapy [5, 29–31]. Consistently, our data further validated that elderly group were less likely to receive any treatment than younger group, although patients can still get survival benefit from surgery and adjuvant therapy even with distant metastases [32, 33]. This was probably due to concerns about increased comorbidities that induce decreased treatment tolerability [34]. In addition, our study also identified various independent prognostic factors for MBC patients aged over 80 years. For example, we demonstrated that patients with white race or married status were significantly related with increased CSS, which could partly due to socioeconomic factors [35]. We also found that patients with HR+/Her2+ subtype had a better prognosis than those with HR+/Her2-. We suspect that the recent great advance in anti-Her2 targeted therapy may account for it [36].

Inevitably, several potential limitations may exist in this study. Firstly, this analysis is limited by selection bias due to intrinsic weaknesses of retrospective study. Secondly, metastatic data in SEER database is restricted to five organs (distant lymph node, bone, liver, lung and brain) and other distant metastasis sites are unclear. Thirdly, detailed information such as co-morbidities, endocrine therapy, and anti-Her2 targeted therapy are limited in the SEER database, which are thought to be closely related to patients’ survival. Hence, our results need to be further validated in future studies.

## Conclusion

In summary, our study summarized the metastasis pattern and survival outcome of MBC patients aged over 80 years from a large sample of the population. We found that MBC patients with different age presented with distinctive metastatic patterns, clinical characteristics, and prognostic outcomes. Our findings could provide a better understanding of clinical features in elderly MBC patients and may assist clinicians to make appropriate treatment.

## Supporting information

S1 Table Univariate analysis and multivariate analysis for overall survival (OS) and cancer specific survival (CSS) among patients with metastatic breast cancer. (DOCX)
