# Supplementary material for: Metastasis patterns and prognosis of octogenarians with metastatic breast cancer: A large-cohort retrospective study
Source: PLoS One. 2022 Feb 17;17(2):e0263104. doi: 10.1371/journal.pone.0263104 (PMC8853583; doi:10.1371/journal.pone.0263104)
Supplement: S1 Table — (DOCX) [file pone.0263104.s001.docx]

Table S1. Multivariate analysis for overall survival (OS) and cancer specific survival (CSS) among patients with metastatic breast cancer.

|  | OS | |  | CSS | |
| --- | --- | --- | --- | --- | --- |
| Variables | HR (95% CI) | P value |  | HR (95% CI) | P value |
| Age |  | <.001 |  |  | <0.001 |
| <35 | Reference |  |  | Reference |  |
| 35-49 | 1.13 (0.96-1.32) | 0.142 |  | 1.13 (0.96-1.34) | 0.133 |
| 50-79 | 1.39 (1.20-1.62) | <.001 |  | 1.34 (1.14-1.56) | <.001 |
| >79 | 2.11 (1.78-2.50) | <.001 |  | 1.89 (1.58-2.26) | <.001 |

Abbreviations:

HR: Hazard ratio.
